# A Propensity-Matched Cohort Study Assessing Neuropathy in Patients With Leukocytoclastic Vasculitis

**DOI:** 10.7759/cureus.87177

**Published:** 2025-07-02

**Authors:** Kritin K Verma, Sezim Minbaeva, Orson T Robertson, Ryan Koch, Daniel P Friedmann, Denise C Robson, Jonathan Aldrete, Michelle Tarbox

**Affiliations:** 1 School of Medicine, Texas Tech University Health Sciences Center, Lubbock, USA; 2 School of Medicine, Texas A&M University, College Station, USA; 3 Westlake Dermatology Clinical Research Center, Westlake Dermatology and Cosmetic Surgery, Austin, USA; 4 Dermatology, Texas Tech University Health Sciences Center, Lubbock, USA

**Keywords:** immune complex, leukocytoclastic vasculitis, neuropathy, risk ratio, systemic involvement

## Abstract

Objective

The objective of this study was to examine the potential relationship between leukocytoclastic vasculitis (LCV), a small-vessel vasculitis, and neuropathy. While LCV primarily affects the skin, its systemic involvement, including the development of neuropathy, has been increasingly recognized. This study aimed to assess whether LCV patients have a higher risk of developing neuropathy compared to a demographically matched control group.

Materials and methods

This retrospective cohort study utilized data from the TriNetX database (TriNetX, LLC, Cambridge, Massachusetts, USA). A total of 4,519 patients diagnosed with LCV were matched with 4,519 control patients based on demographic factors such as age, sex, and race to ensure comparable baseline characteristics between groups. Neuropathy in both groups was identified using the ICD-10-CM diagnostic code G62.9. Statistical analysis was performed to evaluate the relative risk of neuropathy in LCV patients compared to the control group.

Results

The analysis revealed that 5.4% of LCV patients were diagnosed with neuropathy, while 4.6% of the control group were affected. This difference was statistically significant, with a relative risk (RR) of 1.209, indicating that LCV patients have a 20.9% higher risk of developing neuropathy than controls (95% confidence interval (CI): 1.009-1.448, p = 0.0396). These findings suggest that LCV may contribute to the development of neuropathy.

Conclusion

This study provides evidence supporting the association between LCV and an increased risk of neuropathy. The findings highlight the potential for systemic neurological complications in patients with LCV, which may be attributed to vasculitic damage to the nerve blood supply. Despite the challenges in diagnosing LCV-associated neuropathy, the study underscores the importance of vigilant monitoring for neurological symptoms in LCV patients.

## Introduction

Leukocytoclastic vasculitis (LCV) is a type of small-vessel vasculitis that most classically results from an Arthus-type III hypersensitivity reaction, in which antigens complexed to IgG or IgM deposit in the walls of small blood vessels [1]. These immune complexes activate the complement system, leading to neutrophil infiltration and subsequent inflammation and damage to the vessel walls [2,3]. Clinically, LCV typically presents as palpable purpura and may rarely demonstrate urticarial lesions [1]. Histopathological examination is crucial for diagnosis and is marked by the presence of neutrophil infiltrate, often accompanied by fibrinoid necrosis of the vessel walls [1,3]. While mostly affecting the skin, systemic involvement, including neuropathy, is becoming more widely recognized [4,5]. In recent years, there has been an increasing awareness of the broader clinical implications of LCV, particularly its association with various neurological disorders [6], including peripheral neuropathy and central nervous system involvement [4,5]. Literature suggests that neuropathy in LCV may present in various forms, from sensory disturbances (e.g., paresthesia) to motor impairment, with some cases progressing from sensory to motor disturbances, highlighting the diverse manifestations of this condition [7,8]. This suggests that LCV is not only a dermatological concern but also potentially a multi-system disorder, making it critical to explore its broader impact. This study employs the TriNetX database (TriNetX, LLC, Cambridge, Massachusetts, USA), leveraging its large-scale real-world data, to investigate the relationship between LCV diagnosis and neuropathy [9,10]. Further research is needed to better understand this relationship, understand its underlying mechanisms, and develop targeted therapies that can address both the vascular and neurological components of the disease [5,9,11]. 

## Materials and methods

Study design

**Figure 1:**
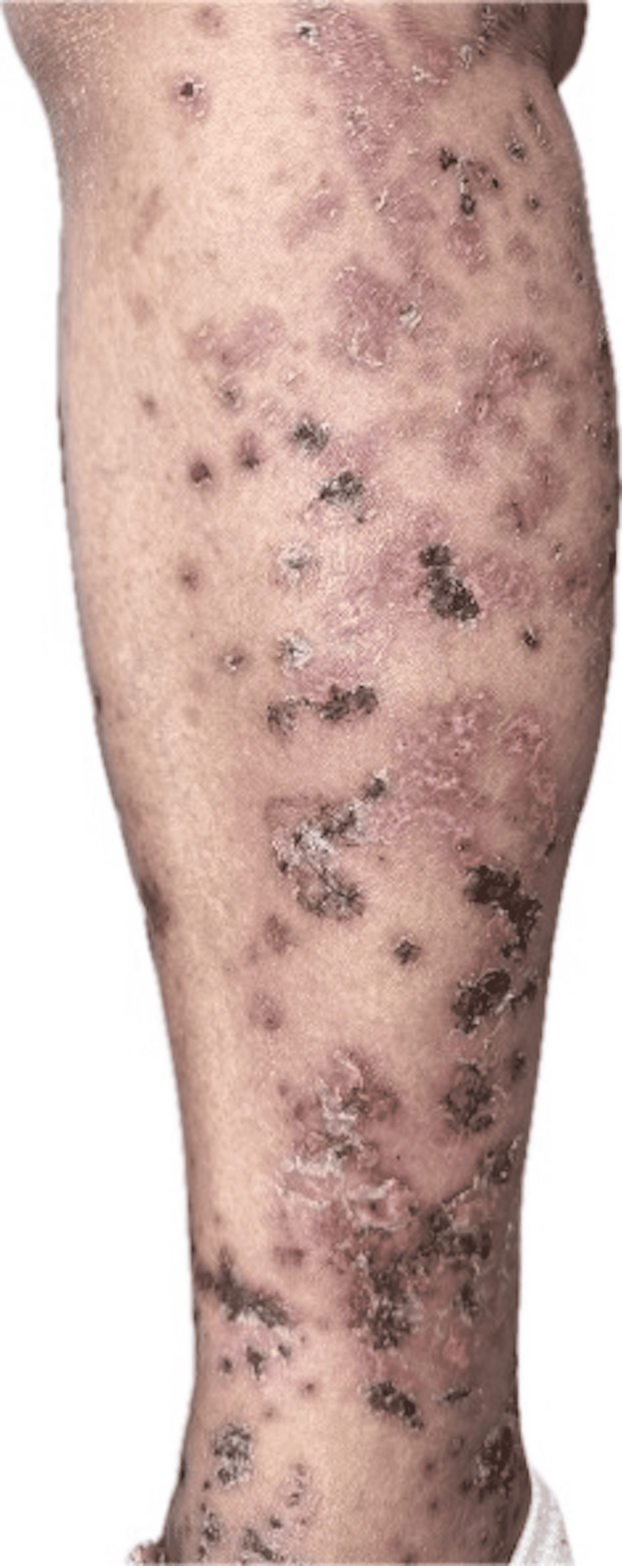
Leukocytoclastic vasculitis visualized on the anteromedial aspect of the right lower leg. Image source: Original photograph contributed by Michelle Tarbox and Denise Robson (co-authors of this manuscript).

This was a multi-institutional study conducted using retrospective, de-identified patient data from the TriNetX Research Network, which aggregates electronic medical records from multiple healthcare organizations across the United States. We conducted a retrospective cohort study using the TriNetX database to identify cases of LCV (Figure 1). The study was conducted in February 2025, during which we identified 4,519 instances of LCV using the ICD-10-CM codes D69.0 (LCV) and L95.8 (cutaneous LCV), analyzing the past 20 years of data [12]. To minimize potential confounding factors, we employed propensity score matching to select control subjects. The controls were matched by age, sex, and race using optimized algorithms available within the TriNetX platform [3].

Inclusion and exclusion criteria

Inclusion criteria for this study were individuals with a diagnosis of LCV identified by ICD-10-CM codes D69.0 and L95.8. Exclusion criteria involved removing individuals who did not meet the matching criteria (age, sex, and race) or those without sufficient data within the TriNetX database to conduct a complete analysis [3].

Data collection

Data was collected from the TriNetX database, which includes a wide range of demographic information as well as diagnostic codes [3]. Neuropathy cases were identified through the ICD-10-CM code G62.9 (polyneuropathy, unspecified). This code was specifically chosen because other forms of neuropathy, such as diabetic or alcoholic neuropathy, would not fit into the category of LCV-induced neuropathy. We focused on collecting demographic data and the prevalence of neuropathy in patients with LCV compared to the control group.

Race and ethnicity data within the TriNetX platform are collected and recorded by participating healthcare organizations during routine clinical care. This information is typically self-reported by patients at the time of registration or intake and entered into electronic health records by clinical or administrative personnel. The data is de-identified and aggregated for research use within the TriNetX platform.

Statistical analysis

Demographic data and the prevalence of neuropathy were analyzed using Wald's method. We calculated risk ratios (RRs) and 95% confidence intervals (CIs) to assess the association between LCV and the development of neuropathy. Logistic regression analysis was used in congruence with Wald's method. Statistical significance was determined using a threshold of p < 0.05.

## Results

The final cohort of 4,519 LCV patients and 4,519 matched controls was well-balanced in terms of sex (51% female in both groups) and age (mean 43.3 ± 22.9 years), with a similar racial distribution (68% White individuals in both groups). This careful matching strengthens the validity of the comparison by minimizing demographic confounding. The key observation was that neuropathy occurred in 5.4% (243/4,519) of LCV patients versus 4.6% (206/4,519) of controls, resulting in a relative risk (RR) of 1.209 (95% CI: 1.009-1.448; p = 0.0396) (Table 1).

**Table 1:** Propensity-matched cohort analysis of demographics and neuropathy in patients with leukocytoclastic vasculitis from the TriNetX database. *After propensity score matching, **Statistically significant values between cases and controls, defined at p < 0.05 using Wald's statistical test CI: confidence interval; CM: clinical modification; ICD: International Classification of Diseases; RR: risk ratio; SD: standard deviation

Characteristic	Cases (%)	Controls (%)	RR (95% CI)*	P-value*
Total Participants	4,519 (100%)	4,519 (100%)	-	1.00
Average age ± SD	43.3 ± 22.9	43.3 ± 22.9	-	0.995
Female	2,304 (51.0%)	2,298 (50.9%)	-	0.003
Male	2,088 (46.2%)	2,093 (46.3%)	-	0.916
Race				
White	3,071 (68.0%)	3,077 (68.1%)	-	0.892
Alaska Native	23 (0.5%)	19 (0.4%)	-	0.536
Native Hawaiian	23 (0.5%)	22 (0.5%)	-	0.881
Black	332 (7.3%)	334 (7.4%)	-	0.936
Asian	157 (3.5%)	153 (3.4%)	-	0.817
Ethnicity				
Non-Hispanic	3,214 (71.1%)	3,216 (71.2%)	-	0.963
Hispanic	603 (13.3%)	602 (13.3%)	-	0.975
Polyneuropathy, unspecified (ICD-10-CM Code: G62.9)	243 (5.4%)	206 (4.6%)	1.209 (1.009-1.448)	0.0396**

The RR of 1.209 indicates a 20.9% increased risk of neuropathy in LCV patients compared to controls. The 95% confidence interval (1.009-1.448) is particularly important because it excludes 1.0 at the lower bound, reinforcing that the risk elevation is not due to chance. 

The p-value (0.0396) confirms statistical significance while suggesting that replication in larger cohorts could help strengthen these findings, but clinical relevance depends on context. Neuropathy, even at a modestly increased risk, can have meaningful consequences, such as chronic pain, sensory deficits, or mobility issues, particularly in patients already managing systemic inflammation from LCV. Since LCV is a vasculitic disorder, microvascular damage could contribute to nerve ischemia, potentially explaining the observed association.

The findings suggest that LCV patients may benefit from periodic neurological assessments, especially if they exhibit other risk factors (e.g., diabetes, alcohol use, or nutritional deficiencies that independently predispose to neuropathy). 

The study’s matched design reduces confounding, but unmeasured variables (e.g., comorbidities, disease severity, or treatment differences) could influence the results. Future research could explore whether neuropathy in LCV patients follows a distinct pattern or whether tighter inflammatory control mitigates risk.

## Discussion

The findings indicate a significant association between LCV and the development of peripheral neuropathy. This aligns with prior studies that have shown LCV, although classically presenting with skin manifestations, may extend past cutaneous symptoms to encompass neurological complications [5,11]. Neuropathy in LCV is thought to originate from ischemic damage produced by vasculitis inflammation of the vasa nervorum, the vessels which supply the peripheral nerve circulation [13,14]. Mononeuritis multiplex and polyneuropathy have been reported as severe symptoms of systemic vasculitis, with the latter uniquely presenting following recurrent tuberculosis infection [4,7,15].

Several mechanisms may underlie the connection between LCV and neuropathy [14]. Immune complex deposition in small arteries can cause inflammation, luminal narrowing, and ischemia in peripheral nerves and their blood supply [13,14]. Inflammatory responses are further intensified by the release of pro-inflammatory cytokines and other mediators, which may cause nerve damage and worsen vascular damage [13]. Although LCV is often linked to the skin and commonly manifests as palpable purpura [15], systemic involvement is not uncommon and may include abdominal pain, hematuria, and signs of peripheral nerve involvement [4].

The diagnosis of LCV-associated neuropathy can be difficult and may necessitate a combination of clinical examination, electromyography, nerve conduction investigations, and, in some cases, biopsy of affected tissues [4,6,8]. Skin biopsy may be especially beneficial to rule out medium and/or large vessel vasculitis [13].

LCV-associated neuropathy is often treated with immunomodulatory treatments to reduce the underlying vascular inflammation. Corticosteroids are frequently utilized as first-line treatment, and long-term maintenance may necessitate "corticosteroid-sparing" agents. In some patients, intravenous immunoglobulins or plasma exchange may help with acute exacerbations.

Limitations of this study include the reliance on ICD-10 codes for case identification, which may have resulted in some misclassification [16], as LCV diagnoses may have been categorized into broader categories or overlooked entirely due to coexisting conditions [6]. Furthermore, although varying degrees of neuropathy have been observed throughout the literature, the analysis makes no distinction between the types or degrees of neuropathy, which may affect the strength of the observed association [11]. Despite propensity-score matching, residual confounding from characteristics such as comorbid autoimmune diseases, medication use, or other unmeasured variables remains a possibility [15,17,18]. The TriNetX database, although reflective of real-world clinical practice, may not capture all relevant clinical details, and its use of broad diagnostic criteria may limit the accuracy and generalizability of the findings [6,10]. These limitations highlight the need for further research to refine diagnostic criteria and explore the mechanisms of neuropathy in LCV [6].

## Conclusions

Although diagnosing LCV-associated neuropathy can be challenging, the study highlights the need for careful monitoring of neurological symptoms in patients with LCV. This study found a weak but statistically significant link between LCV and neuropathy, reinforcing the need for clinical awareness. Future research should concentrate on investigating this relationship whilst accounting for additional variables (e.g, comorbidities known to cause peripheral neuropathy). 
